# Data-driven tailoring of molecular dipole polarizability and frontier orbital energies in chemical compound space†

**DOI:** 10.1039/d3cp02256k

**Published:** 2023-08-11

**Authors:** Szabolcs Góger, Leonardo Medrano Sandonas, Carolin Müller, Alexandre Tkatchenko

**Affiliations:** a Department of Physics and Materials Science, University of Luxembourg L-1511 Luxembourg City Luxembourg alexandre.tkatchenko@uni.lu

## Abstract

Understanding correlations – or lack thereof – between molecular properties is crucial for enabling fast and accurate molecular design strategies. In this contribution, we explore the relation between two key quantities describing the electronic structure and chemical properties of molecular systems: the energy gap between the frontier orbitals and the dipole polarizability. Based on the recently introduced QM7-X dataset, augmented with accurate molecular polarizability calculations as well as analysis of functional group compositions, we show that polarizability and HOMO–LUMO gap are uncorrelated when considering sufficiently extended subsets of the chemical compound space. The relation between these two properties is further analyzed on specific examples of molecules with similar composition as well as homooligomers. Remarkably, the freedom brought by the lack of correlation between molecular polarizability and HOMO–LUMO gap enables the design of novel materials, as we demonstrate on the example of organic photodetector candidates.

## Abstract 2

Using the extended QM7-X dataset, it is shown that polarizabilty and HOMO–LUMO gap are uncorrelated in the chemical compound space. The lack of correlation enables the design of novel materials, demonstrated through organic photodetector candidates.

## Graphical abstract

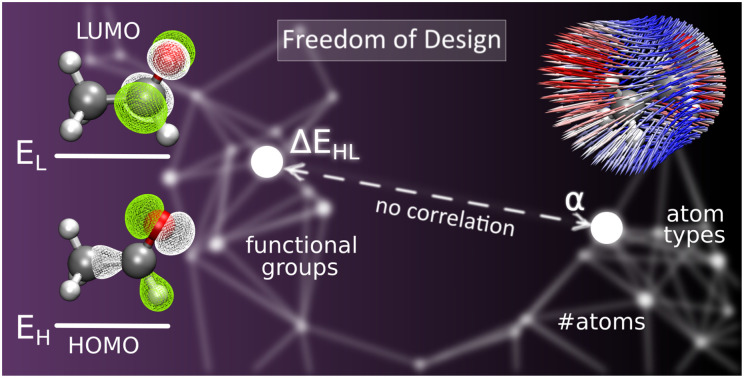

## 1 Introduction

Data-driven molecular design is an increasingly pursued strategy in chemical physics and computational chemistry. The search for novel molecules with tailored physicochemical properties for a given functionality is continuously motivating the development of a great variety of computer-aided molecular design approaches.^1–3^ The ultimate goal is to establish a feasible protocol that can be used for exploring the chemical compound space (CCS) through systematic targeting of physical properties. Physicochemical quantities, such as color, conductivity, excited state lifetime, electron affinity, ionization potential, and solubility, are commonly used in the design of molecular photosensitizers or optoelectronic devices, for instance.^4–7^ Given the complexity of a multi-property design task, it is essential to first have a solid grasp of the physical relationships between the various target properties.^8^

Within this context, two fundamental quantum-mechanical (QM) electronic properties are the optical gap and the molecular dipole polarizability (*α*). Optical gap is an experimental property that measures the energy corresponding to the lowest observed optical transition. Many computational studies use the HOMO–LUMO gap Δ*E*_HL_ (the difference between the energies of frontier molecular orbitals in the ground state) as a starting point in approximating experimental optical gaps. This approximation is widely favored due to the computational challenges associated with employing highly accurate quantum mechanical methods incorporating orbital relaxation effects (*e.g.* time-dependent density functional theory or multi-configurational self-consistent field methods), especially, when investigating vast areas of the CCS, macromolecules, molecular aggregates, or molecular junctions.^9–11^ Thus, the HOMO–LUMO gap plays a crucial role in understanding various aspects of chemical reactivity, excitation energies, and several key optical properties in these organic systems. For instance, its calculation is essential for gaining insights into optical absorption spectra, refractive indices, and conductivity.^12–15^ For correctness of terminology, HOMO–LUMO gap obtained from density functional calculations should be referred to as Kohn–Sham (KS) gap. Although the relations between different gaps (Kohn–Sham, fundamental, and optical) are subtle and have been discussed in detail in the literature,^16,17^ in this manuscript we will use the KS gap as a proxy for observable experimental properties.

The molecular dipole polarizability *α* (referred to simply as polarizability in the manuscript), on the other hand, describes the dipolar response of a molecule to an external electric field, becoming a key quantity for understanding intra- and intermolecular interactions (*e.g.* dispersion interactions, substituent and solvent effects as well as supramolecular structure formation) and for determining spectroscopic properties of molecules (Raman, Raman optical activity and sum frequency spectroscopy).^18–25^ These features make both polarizability and HOMO–LUMO gap essential in the derivation of structure–property/property–property relationships and, consequently, in the development of design strategies for molecules with a targeted array of QM properties for applications such as molecular dyes,^26^ optoelectronic devices,^27^ molecular junctions,^28,29^ heterogeneous catalysts^30^ and materials for non-linear optics.^31,32^

Various computational methods and predictive models have been developed to estimate HOMO–LUMO gaps and polarizabilities for organic molecules with different levels of tradeoff between accuracy and computational cost.^33–36^ Lately, it has became feasible to access a plethora of highly-accurate QM properties – including Δ*E*_HL_ and *α* – for large swaths of the chemical compound space (CCS).^37–41^ Comprehensive analyses of these extensive datasets may help to understand the deeper physical picture behind the inherent property–property relationships. With this motivation, we herein perform an exhaustive investigation of the two-dimensional space defined by HOMO–LUMO gap and polarizability (*i.e.* (Δ*E*_HL_,*α*)-space) for small organic molecules with the aim to gain insights into the intrinsic relationship between these two properties. We find that while correlation might appear in homologous molecules (that is, molecules differing by a constant increment meaning that their physicochemical properties follow a general trend), if a large enough subspace of CCS is considered, HOMO–LUMO gap and polarizability are essentially uncorrelated and their 2D space is represented as a structureless “blob”. Through the analysis of diverse molecular sets, it is shown that this lack of correlation can be related to the fact that the polarizability is primarily determined by the atomic composition, while the HOMO–LUMO gap heavily depends on the arrangement of the atoms into chemical functional groups. Hence, we expect that our findings will assist the development of novel design principles in which the control of multiple electronic properties is relevant, as we finally demonstrate on the case of molecular photodetectors.

The outline of the paper is as follows: in Section 2, we review accurate and approximate models for polarizability and HOMO–LUMO gap. In Section 3, we exhaustively examine the polarizabilities (*α*) and HOMO–LUMO gaps (Δ*E*_HL_) of diverse molecular sets. In doing this, we have extended the QM7-X dataset^41^ with functional group information as well as polarizabilities calculated with the hybrid PBE0 functional. In assessing our computational setting, we tested the predictive power of this functional against coupled cluster LR-CCSD calculations, and found an overall accuracy of 1.9%. As a first order approximation to predicting polarizabilities of small organic systems, we consider a linear combination of atomic contributions in Section 3.1. In Section 3.2, we then perform PBE0 calculations of polarizability (see Section 6.1 for computational details) for homologous molecules and explore the relationship with their HOMO–LUMO gaps. A statistical analysis of the (Δ*E*_HL_,*α*)-space using a subset of molecules contained in QM7-X dataset is carried out in Section 3.3. Our proposed design principle is further discussed and demonstrated on the case of organic photodetectors, see Section 4. The computational methods as well as the dataset used are presented in Section 6, following the main conclusions of the manuscript in Section 5.

## 2 Models for polarizability and frontier orbital energy gap

Since our main focus is to have a better understanding of the relationship between polarizability and HOMO–LUMO gap in organic molecules, we first revisit different qualitative and quantitative models employed to compute them. In general, a variety of electronic structure methods can be used to calculate both of these quantities. The choice of a computational level depends on the specific target property and the necessary tradeoff between computational cost and accuracy. While calculating the HOMO–LUMO gap is feasible using various mean-field electronic structure methods, orbital relaxation effects play a significant role in determining optical properties.^42^ However, in computationally expensive studies such as the analysis of macromolecules or extensive datasets (*e.g.* QM7-X considered in this manuscript), HOMO–LUMO gap is often used as a first approximation to experimental quantities. Polarizability (*α*) is typically obtained from finite field, coupled perturbed Hartree–Fock or density functional perturbation theory (DFPT) calculations.^33,34,43^ However, these electronic structure methods need considerable computational resources when dealing with larger molecules or significant swaths of the CCS. Accordingly, we will next discuss alternative physical models, empirical correlations, and approximate methods that can be used to obtain these QM properties as well as to broaden the comprehension of property–property relationships in CCS. We will start with examining the polarizability, for which analytical models (such as the quantum Drude oscillator, or QDO) as well as empirical correlations and predictive semiempirical methods are available. After this, the models for HOMO–LUMO gap will be mentioned, before concluding the section by analyzing what is known about the correlation between these two quantities.

A connection between HOMO–LUMO gap and polarizability can be anticipated starting from the perturbative expression for polarizability using the dipole moment operator *μ̂* within second order perturbation theory as^20,44^1
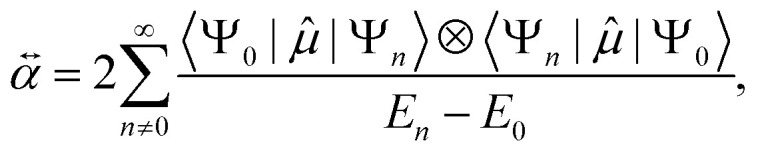
where *Ψ*_0_ and *E*_0_ are the ground state wavefunction and energy, respectively, and *n* is the index of the excited states. Indeed, since Δ*E*_HL_ = *E*_1_ − *E*_0_ is commonly much smaller than the energy gap of higher excited states, the first term of the sum in eqn (1) provides a first-order approximation to the infinite series and, hence, there could exist an inversely proportional relationship between Δ*E*_HL_ and *α*, *i.e. α*∝ (Δ*E*_HL_)^−1^.

Eqn (1) can only be analytically evaluated for simple model systems (such as the hydrogen atom or a quantum Drude oscillator). For many-electron systems, the sum can only be evaluated numerically and requires including bound-bound and bound-continuum transition dipoles.^45^ Modeling atoms or larger coarse-grained fragments with QDOs and solving the dipole–dipole screening equations is known to be an effective method to predict polarizability, and it is also the basis for the Many-Body Dispersion (MBD) method.^46,47^ Since the response properties of all atoms and molecules can be represented by QDOs by carefully setting the three parameters {charge *q*, frequency *ω*, mass *μ*} of the model, the analysis of the polarizability of the QDO Hamiltonian should generally be transferable to any systems. Therefore, we devote some attention to this model.

Due to selection rules of the dipole operator, only the first excited state contributes to the dipole polarizability of a QDO,^44,48^ making it effectively a two-state system2
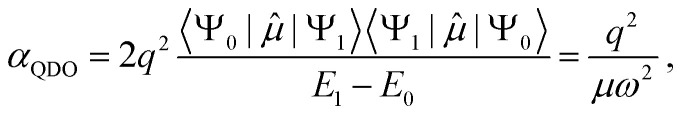
where *q* is the magnitude of the charge bound by a harmonic potential with frequency *ω*, having a mass *μ*. The HOMO–LUMO gap of a QDO in atomic units is Δ*E*_HL_ = *ω*, which indeed appears in the denominator. However, *α* can be separately controlled through the other two individual QDO parameters {*q*,*μ*}, independently from Δ*E*_HL_. This means that for the QDO model, the polarizability and the HOMO–LUMO gap are mutually related, yet they could be tuned separately from each other.

The idea of approximating the polarizability using an effective two-state system (so-called Unsøld approximation)^44,49^ is also useful for understanding qualitative trends. Within this approximation, polarizability is written using an average excitation Δ*E* as a fitting parameter3
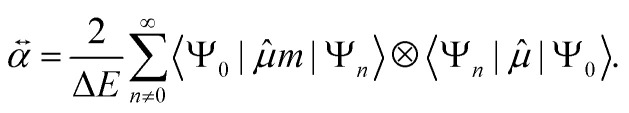
Setting the average excitation to Δ*E*_HL_ is therefore exact for the QDO model, but the connection between these quantities for many-electron systems is not known in general.^50^

Investigating correlations between polarizability and various molecular properties can lead to useful relationships such as the recent observation that polarizability scales with the fourth power of the characteristic size of the system.^44^ The correlation between polarizability and orbital energies is relevant from a theoretical standpoint, as it forms the foundation of Pearson's hard-soft acid–base (HSAB) theory.^51,52^ Based on recent theoretical works, we can postulate that polarizability can be expressed as a function of two factors accounting for (i) ground state geometry (*e.g.* van der Waals radius or molecular volume) and (ii) electronic structure (*e.g.* ionization energy or hardness).^31,44,53–58^ While these correlations provide useful conceptual insights, they have not been put to use for constructing accurate numerical predictions.

There are two types of predictive models for polarizability with lower computational burden than electronic structure calculations. Firstly, approximations for polarizability can be constructed based on the group contribution principle, which divides polarizability into atomic or bond contributions.^30,59^ These models can offer somewhat accurate predictions with minimal molecular information and computational effort, and we will assess such models in this work. As a second approach, machine learning (ML) models have been proposed as a cost-effective solution with improved accuracy.^35^ However, the training process and accuracy of the ML models are strongly dependent on the features of the dataset (*e.g.* chemical diversity, molecular size, number of samples) as well as on the ML method itself.

For the case of HOMO–LUMO gap, there is a well-established underlying physical principle in determining this property: it is known that the HOMO–LUMO gap of individual functional groups (called chromophores in this context) is transferable, with values documented in standard reference texts.^60^ These chromophores also form the foundation for both accurate ML models and earlier empirical rules for the prediction of HOMO–LUMO gaps.^61,62^ The HOMO–LUMO gap of a single functional group can be understood based on the molecular orbital theory, the most common version of which is the Hückel theory for conjugated systems. For instance, the inverse proportionality between the number of monomers and the HOMO–LUMO gap of polyenes is well-explained within this theory.^63^ In the case of non-interacting functional groups, their optical spectra are effectively independent and, consequently, the frontier energy gap of a molecule is determined by the lowest value for the constituent functional groups, making it an inherently size-independent (intensive) property.

In agreement with the analysis of the QDO model, recent studies relying on large datasets (7 k structures from the GDB-13 dataset as well as the tmQM dataset of 86 k transition metal complexes) suggest that there's no overall correlation between HOMO–LUMO gap and polarizability.^64,65^ Nevertheless, correlation has been observed both experimentally and computationally for different classes of structures (*e.g.* organic dyes and inorganic clusters^26,66–68^), with notable exception of smaller systems where the HOMO–LUMO transition is symmetry forbidden.^24^ In the following section, we explore the source of such seemingly contradictory results by showing that investigating a reduced subset of the chemical compound space can lead to correlations between quantities that are generally uncorrelated.

## 3 Results and discussion

### 3.1 First order linear atomic additive model for polarizability

To understand the correlation between polarizability and HOMO–LUMO gap, we first show that polarizability can be, up to a large degree, determined by knowing only the atomic composition (*i.e.* the types and the numbers of atoms present in a molecule). This analysis is done using the PBE0 polarizability values calculated for the QM7-X molecules, contrasting these quantum chemical results with a linear atomic additive method. The simplest atomic additive method (motivated by that of Bosque^36^) approximates the polarizability of a molecule *via* a linear combination of the number of each atom-types *n* weighted with a type-specific factor *C*_*i*_, together with an intercept *m*4
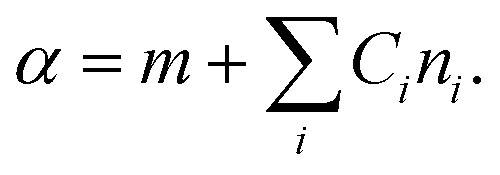


Bosque's model was fit directly using experimental data of 426 compounds. The fitted *m* and *C*_*i*_ values for C, Cl, H, N, O, and S are listed in Table 1. Accordingly, we have here used the QM7-X dataset^41^ (see Section 6) to validate the accuracy and reassess the model parameters on a significantly larger swath of the CCS. In doing so, we have considered the first conformer for each entry in the QM7-X dataset; a total of ≈13 k structures. The linear regression parameters optimized on QM7-X yield the results listed in Table 1. Bosque's parameters hold up relatively well for QM7-X molecules, accounting for a correlation coefficient (*R*^2^ value) of 0.65 with a mean absolute percentage error (MAPE) of 6.11%. However, the re-fitted parameters improve the correlation coefficient to 0.72 and reduce MAPE value to 3.94%, *i.e.* the prediction accuracy is increased by a factor of 1.6. Note that the presence of the intercept *m* in eqn (4) is just an artifact of the model, since the prediction should be zero when no atoms are present. Inclusion or omission of *m*, however, changes neither the goodness of the regression, nor the numerical value of the atomic contributions to a meaningful degree (the mean absolute error of the linear model goes from 3.078 a.u. to 3.079 a.u.; similarly to what had also been observed in the original paper^36^). Therefore, we decided to include the intercept in our further analysis, to be consistent with Bosque's approach.

**Table 1:** Revised linear regression parameters for the atomic additive polarizability model of Bosque *et al.* Note that the values in the original paper are presented in Å^3^, whereas the values here are in Bohr^3^. The relatively low influence of the intercept can be seen by comparing the last two rows; the rest of our manuscript uses the parameters presented under “'This work”

	Intercept	C	Cl	H	N	O	S
Bosque	2.14	10.20	14.60	1.17	6.95	3.85	20.20
This work	1.71	10.10	12.70	0.87	7.88	4.00	19.10
No intercept	0.00	10.37	13.00	0.88	8.11	4.24	19.37

**Fig. 1:**
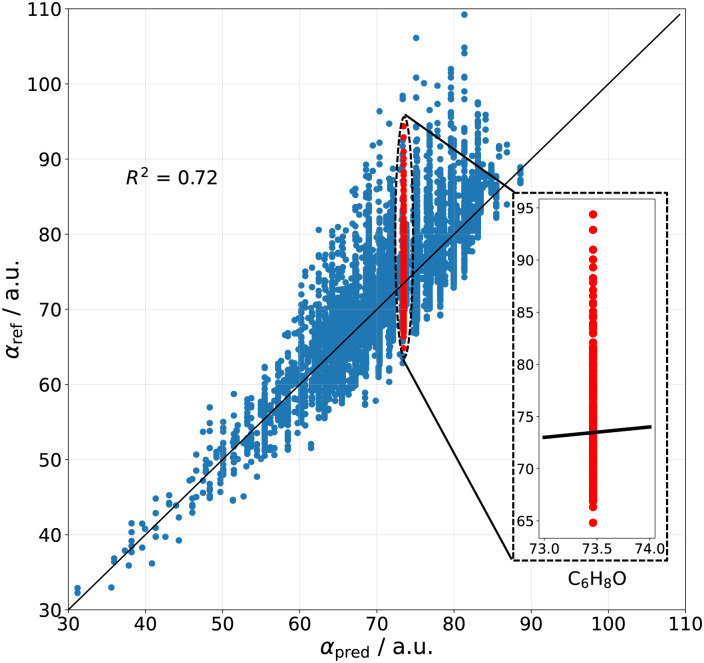
Performance of the atomic additive method. The linear regression parameters are fit utilizing our dataset of 13 k molecules computed at the DFT-PBE0 level of theory (subset of the QM7-X dataset,^41^ see Fig. S3 of ESI†). The inset shows the inherent shortcoming of the model, predicting the same polarizability for all molecules having the atomic composition C_6_H_8_O.

A shortcoming of atomic additive methods is that the same polarizability is predicted for all structural isomers, since only the total number of each atom-types is used in the prediction. This is manifested in Fig. 1 as having a systematic error within each possible *α*_pred_ value, and further demonstrated in the inset on the case of molecules with the atomic composition C_6_H_8_O – the reference polarizabilities for this chemical formula span a range of 30 a.u., but the predicted value is 73.4 a.u. for all molecules, irrespective of the chemical arrangement of the atoms. From Fig. 1, it can also be inferred that such a simple additive model will only become worse for molecules of increasing size. Indeed, a trend appears where larger molecules exhibit stronger deviations towards higher polarizabilities – a trend that an additive model is unable to describe. This can be especially the case for polymeric molecules which form long chains, whose polarizability is highly anisotropic and behaves increasingly non-additively with size.

To differentiate between structural isomers, a descriptor that accounts for different geometric properties (for example, radius of gyration) might be constructed, since polarizability is an extensive property.^55,58^ This extensivity is only partially captured by atomic additive methods, insofar as increasing the number of atoms in a molecule is inherently increasing the size as well. More accurate models should also differentiate between similar atoms based on their surrounding chemical environments, as it is done for example in ref. 44, where the environment is taken into account by Hirshfeld partitioning as well as in the self-consistent screening approach used in the Many-Body Dispersion (MBD) method.^46,47^ Therefore, while the shown first-order linear model is limited by its accuracy, it can serve as a baseline for more accurate methods involving coupling between atoms in a molecule.

To summarize, a first-order approximation to polarizability can be constructed just by using an atomic additive model without explicit knowledge of the molecular spatial arrangement or the local chemical environments. While the predictive power of such a model is rather restricted (*i.e.* the chemical environment of each atom is not described), its rough correlation with reference electronic-structure calculations (see Fig. 1) gives a clear evidence that a significant fraction of the polarizability is determined by just the atomic composition.

### 3.2 Case studies for the relation between the HOMO–LUMO gap and polarizability

Having shown that polarizability depends mainly on the atomic composition of molecules, we now turn into exploring the correlations between polarizability and HOMO–LUMO gap. In doing so, we here discuss a set of case studies of select molecules, with all calculations being done using the PBE0 functional, as described in Section 6.

Experimental studies often focus on examining molecules with similar electronic structures, leading to hidden correlations between optical gap and polarizability. In our path towards the general understanding of the relationship between these QM properties, we now examine two different cases: (i) molecules having the same atom-type composition but slightly different chemical compositions and (ii) molecules with the chemical properties fixed while increasing the system size (*e.g.* oligomers).

#### Constitution isomers

In general, the functional groups in a molecule govern the nature and order of the molecular orbitals, determining the HOMO–LUMO gap and the orbitals involved in the electronic transitions. To explore the relationship between HOMO–LUMO gap and polarizability as a function of chemical functionality, we present select examples of constitutional isomers, *i.e.* molecules with the same atomic composition that belong to different substance classes due to the presence of different functional groups.

**Fig. 2:**
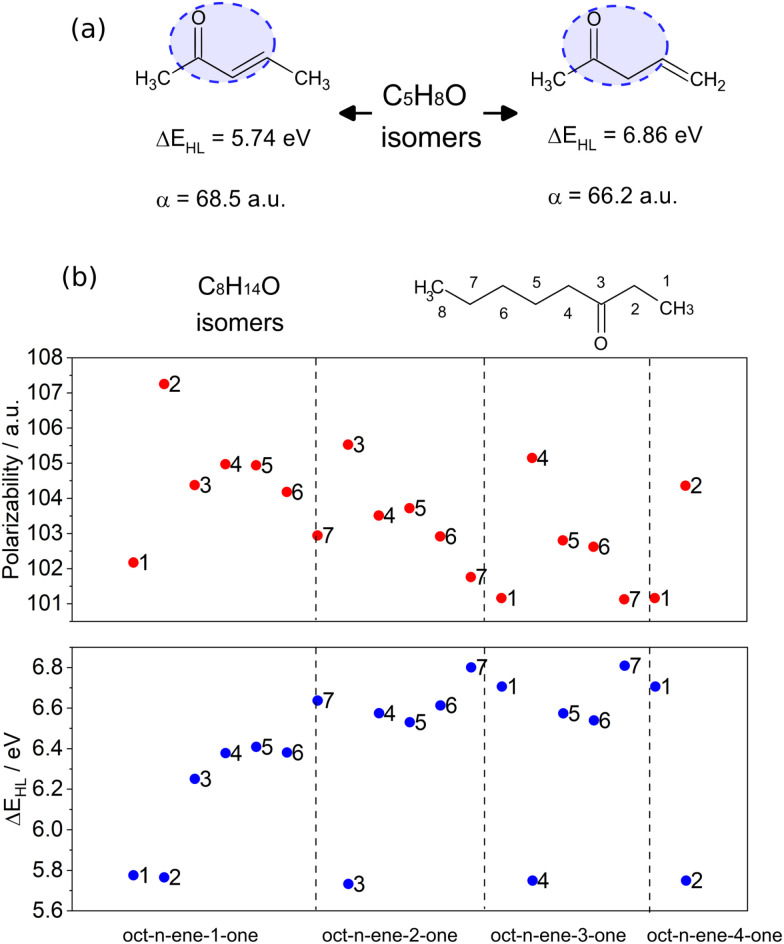
(a) Two constitution isomers, *i.e.* molecules with the same atomic composition but different chemical properties, showing similar polarizabilities but different HOMO–LUMO gaps. (b) HOMO–LUMO gap and polarizability of all possible linear structures having eight carbon atoms, an oxo group and a double bond between two of the carbons. The numbering of carbon atoms is shown on octane-3-one, with *n* representing the numbering of the carbon atom at the start of the double bond (see Fig. S5 of ESI,† for the explicit structures). The polarizability and HOMO–LUMO gap values are results of PBE0 calculations as described in Section 6.1.

As a first example, two constitutional isomers with the formula C5H8O, namely an α,β-(3-penten-2-one) and a β,γ-unsaturated enone (4-penten-2-one) is considered (see Fig. 2(a)). It is noticeable that 3-penten-2-one has a smaller gap compared to 4-penten-2-one (by 1.12 eV) because delocalization results in a greater mobility of π-electrons throughout the molecular structure. However, both molecules present a similar polarizability coming from the identical atomic composition as well as a similar total size.

A second set of constitutional isomers with the formula C_8_H_14_O was constructed for molecules bearing a C=O (oxo-group) and C=C (alkene-group) on an octane backbone. These isomers are thus formed by the following substance classes: one ketene, one conjugated aldehyde, four conjugated ketones, five non-conjugated aldehydes and eight non-conjugated ketones (see Fig. 2(b) as well as Fig. S5 of ESI†). While these structures are chemically quite different, their orbital symmetries are largely similar, leading to a correlation between their polarizability and HOMO–LUMO gap. Notice, however, that the polarizabilities of the structures are all within 4% of each other, whereas the variation of HOMO–LUMO gap is about five times larger. As such, the statement that polarizability is mainly determined by the atomic composition and HOMO–LUMO gap by the chemical composition seems to hold, even though some correlation between these two quantities is observed due to the similarity of the structures.

#### Homologous series of molecules

**Fig. 3:**
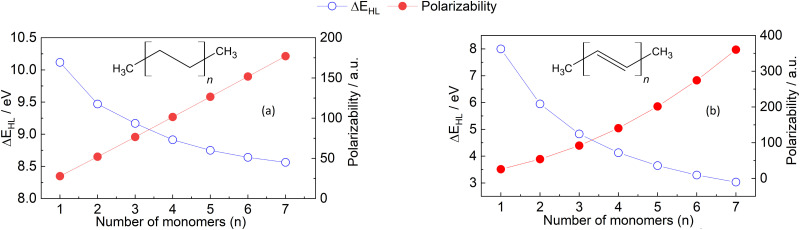
HOMO–LUMO gap (blue dots) and polarizability (red dots) of the oligomers of (a) ethylene and (b) acetylene. The calculations of both properties were carried out as described in Section 6.1.

As previously elaborated, HOMO–LUMO gap and polarizability can seemingly correlate for molecules that belong to a homologous series. This can be explained by the fact that the electronic nature and order of the frontier orbitals is often identical for structurally and electronically similar molecules. Consequently, the decrease in the HOMO–LUMO gap can correlate with the increase in polarizability when considering molecules of a homologous series with an increasing number of repeating units. To support this assumption, we consider in the following a series of oligomers, namely alkanes (C_*m*_H_2*m*+2_) and alkenes (C_*m*_H_2*m*_; see Fig. 3). The example is taken from Afzal *et al.*,^69^ with polarizability and HOMO–LUMO gap recalculated within our computational setup (*cf.* Section 6).

Fig. 3 shows a decreasing behaviour of HOMO–LUMO gap for oligoethylene and oligoacetylene as a function of the number of monomers *n*, in agreement with previous works as well as qualitative predictions from the Hückel model.^63,70^ Indeed, we have found that the absence of a qualitative change to the electronic structure within the ethylene oligomers leads to a relatively small HOMO–LUMO gap change going from *n* = 1 → 7 (≈1.5 eV) compared to the acetylene oligomers, where every monomer modifies the conjugation, producing a more significant change of ≈5.0 eV. Unlike HOMO–LUMO gap, the behavior of polarizability in the molecular chains can not be simply explained. The observation that polarizability monotonously increases with *n* is in line with both the principles of atomic additive models and the correlation with molecular size. However, the absolute magnitude of the polarizability values is significantly different for the two sets of oligomers, and this difference increments with increasing the number of monomers. This quantitative difference can neither be explained by atomic additive models nor correlations using molecular size, but it correlates with the reductions in HOMO–LUMO gap. Notably, not even AlphaML^35^ can predict this behavior: the model predicts 181 a.u. for oligoethylene and 227 a.u. for oligoacethylene in the *n* = 7 case, with the DFPT results being 177 a.u. and 360 a.u., respectively. The difference due to conjugation is therefore underestimated by a factor of four even when using ML methods, and this error is expected to increase with increasing the chain length. Up to now, we are not aware of any other simple polarizability estimation method that can accurately predict the values in Fig. 3. These findings provide clear evidence that further work is necessary to enhance our understanding and improve and accuracy of computational methods used for calculating polarizability, even for relatively simple molecules such as hydrocarbon oligomers.

### 3.3 Clustering of structures in the (Δ*E*_HL_,*α*)-space

To draw more general conclusions about the relationship between the properties in question, we analyse the two-dimensional (2D) property space defined by HOMO–LUMO gap and polarizability for a selected subset of QM7-X molecules^8^ (see Section 6.2). HOMO–LUMO gap values were taken from the dataset, while polarizability values were recalculated using the computational setup explained in Section 6.1. All previously presented examples might suggest that there is a correlation between HOMO–LUMO gap and polarizability. However, these examples considered similar molecules with respect to their functionality or chemical composition – factors that essentially determine both the HOMO–LUMO gap and polarizability. From optical spectroscopy, it is known that the optical gap is primarily determined by the functional groups in a molecule. This is reflected in characteristic optical gaps (vertical excitation energies of the lowest electronic transitions) per functional groups, *e.g*. the ππ* absorption of an isolated alkene-group as chromophore is between 7.51 and 6.70 eV. Since we are assuming that HOMO–LUMO gap is a good starting point in determining the optical gap of a molecule, it would be expected to find that Δ*E*_HL_ values are also clustered by certain functional groups. In contrast, our analysis has shown that polarizability (*α*) is primarily determined by the atomic composition of a molecule. The QM7-X dataset enables us to study the (Δ*E*_HL_,*α*) relationship more broadly because it covers a considerable number and variety of chemical compounds.

**Fig. 4:**
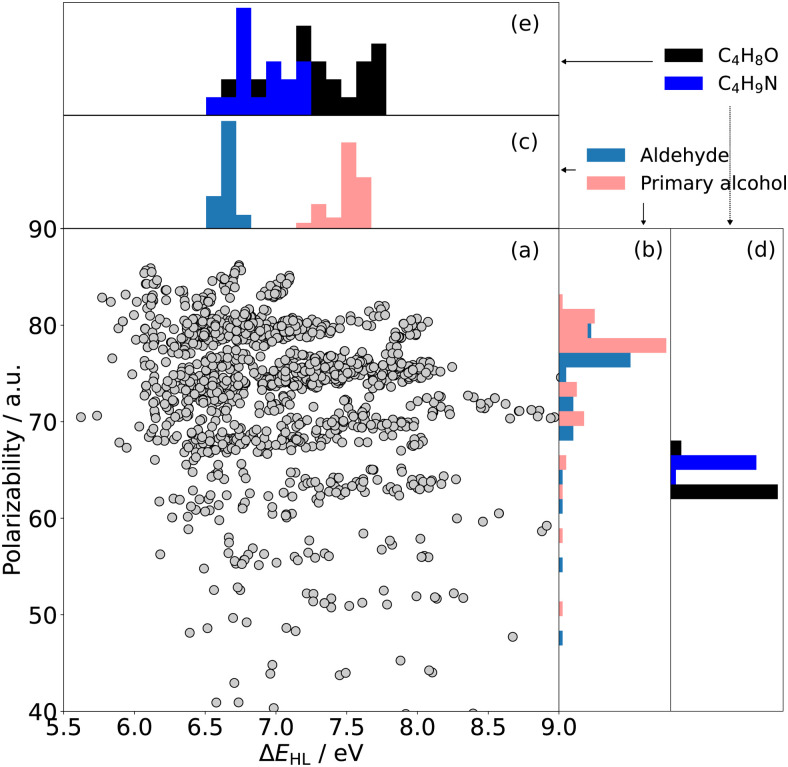
(a) Polarizability (*α*) *vs.* HOMO–LUMO gap (Δ*E*_HL_) for molecules of the subset of QM7-X under study (see text). Histograms of the HOMO–LUMO gaps (b) for all non-conjugated aldehydes (blue) and primary alcohols (pink) and (d) for structures having the atomic composition C_4_H_8_O (black) and C_4_H_9_N (blue). Histograms of polarizabilities (c) for all non-conjugated aldehydes (blue) and primary alcohols (pink) and (e) for structures having the atomic composition C_4_H_8_O (black) and C_4_H_9_N (blue). The difference between the clustering in the two quantities is reflected in the degree of separation between the histograms.

Fig. 4(a) shows the (Δ*E*_HL_,*α*)-space for the QM7-X molecules – indicating no direct relationship between the two quantities across the chemical compound space spanned by this dataset (*R*^2^ = 0.13). Furthermore, the role of the two main factors that determine Δ*E*_HL_ (functionality) and *α* (atomic composition) are highlighted in Fig. 4. The panels (b) and (c) exemplarily display the distributions of Δ*E*_HL_ and *α* for aldehydes and primary alcohols, *i.e.*, molecules that bear one of the respective functional groups. The subplots (d) and (e) show the respective distributions for molecules with equal atomic compositions, namely with the molecular formulas C_4_H_8_O and C_4_H_9_N, respectively.

#### Functional groups & HOMO–LUMO gap

In Fig. 4(b and c), we highlight the frequency plots of Δ*E*_HL_ and *α* values for all non-conjugated aldehydes (blue) and primary alcohols (pink) of our select dataset. Fig. 4(c) clearly reflects the common notion of chromophores, namely that HOMO–LUMO gap is mainly determined by the type of chromophore (*e.g.* aldehyde or primary alcohol group) and the character of the lowest energy electronic transition (*e.g.* nπ*- or nσ*-transition). Thus, Δ*E*_HL_ values for aldehydes only show a value of *circa* 6.5 eV while, for primary alcohol group, they extend from 7.2 eV to 7.8 eV.

To fully explore the role of functional groups on the (Δ*E*_HL_,*α*) relationship, the QM7-X molecules were categorized into twelve major classes based on the functional groups they are bearing (see Section 6.2). Fig. S4 (ESI†) shows that the distribution of all detected functional groups in the dataset, confirming that Δ*E*_HL_ is clustered along the chemical properties of the molecules.

Unlike Δ*E*_HL_, molecules containing aldehydes (blue) and primary alcohols (pink) exhibit polarizabilities that extend throughout the entire range of the dataset (*cf.*Fig. 4(b)). This finding is further reflected in the average Kolmogorov-Smirnov-metric (measuring the statistical distance between two general distributions, see Section II of ESI†) of the individual molecular classes in the (Δ*E*_HL_,*α*)-space, which is 0.81 and 0.40 for Δ*E*_HL_ and *α*, respectively. Our analysis then demonstrates that functional groups primarily affect HOMO–LUMO gap rather than the polarizability, resulting in well-defined molecular clusters on the Δ*E*_HL_-axis.

#### Atomic composition & polarizability

According to the Kolmogorov-Smirnov analysis in Section II of the ESI,† the functional groups only indirectly influence the magnitude of the polarizability in a given molecule, whereas the atomic composition is a crucial factor for the determination of the polarizability. This finding is also in line with the fact that a good correlation is achieved between the first-order atomic additive model and the reference DFT data shown in Fig. 1. The Δ*E*_HL_ and *α* values for a set of two constitution isomers, namely with the chemical formula C_4_H_8_O (including aldehydes, dialkyl ethers, enol ethers, as well as primary and secondary alcohols) and C_4_H_9_N (including carbonitriles and primary/secondary aliphatic amines) is also presented in Fig. 4(d), showing a narrow polarizability distribution. These results are another clear evidence that *α*, to a reasonable approximation, is independent of the actual chemical arrangement of the atoms in the molecule but it mainly depends on the total number of atom-types.

In summary, we can conclude that the lack of overall correlation observed in (Δ*E*_HL_,*α*)-space is a consequence of two main facts: (i) the HOMO–LUMO gap is determined by the nature of the chemical composition (*cf.*Fig. 4c and e) and (ii) the polarizability is largely determined by the atomic composition (*cf.*Fig. 4b and d).

## 4 Case study: design of photodetectors

The possibility of an inverse correlation between the HOMO–LUMO gap and polarizability was thoroughly examined in Section 2. This specific correlation has been observed exclusively when analyzing the electronic properties of homologous series and isomers, as described in Section 3.2. However, when investigating a chemically diverse set of molecules, such as those found in QM7-X, we discovered that these two properties are essentially uncorrelated. This lack of correlation offers a flexibility in the (Δ*E*_HL_,*α*)-space to identify diverse molecules with a targeted pair of properties, *e.g.* by fixing Δ*E*_HL_, one will have a wide range of *α* values where we can select a molecule with desired chemical features. Now, we present how this lack of correlation can be exploited for molecular design purposes. The property data used for this analysis is from the donor–acceptor (DA) dataset,^71^ which was designed to enumerate promising organic photodetector candidate molecules. The DA dataset contains only molecular structures and HOMO–LUMO gap values, while the estimation of polarizability was performed using the revised Bosque model, as elucidated in Section 3.1.

A common challenge in materials science is the effective design of photodetectors. These optoelectronic devices capture light and convert it to electric signal, therefore playing an important role in sensing, monitoring and optical communication. The wide range of physicochemical properties spanned by organic molecules enables various design strategies, which ultimately led to the emerging field of organic photodetectors.^72,73^ HOMO–LUMO gap is one of the key quantities that can be used to approximate the coupling strength of molecules with light, thereby any design strategy motivated by optics will be initially based on this property.^71,74^ Since the fundamental function of photodetectors is to convert light into electrical current, controlling the electrochemical behavior is also crucial. Specifically, the electrochemical work function plays a critical role in the description of organic photodetectors,^75,76^ as opposed to organic semiconductors, where the focus is usually on the charge carrier mobility.^77^ The work function *ϕ* of an electrode is known to change with the polarizability of the absorbed molecules as well as the surface coverage, as described by the Topping equation^78^ (written for a square lattice)5
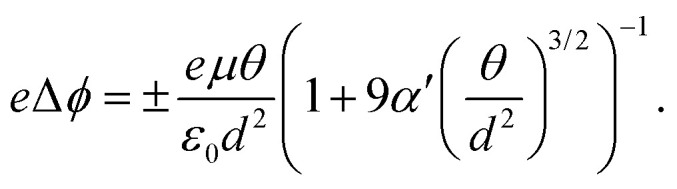
This expression highlights that the work function *ϕ* also depends on the the dipole moment *μ*_0_ and polarizability *α* of the molecules, besides the surface coverage *θ* and the lattice constant of the absorbate *d*. Notice that an effective polarizability *α*′ is used to represent the properties of the absorbed molecules in eqn (5), which is usually an order of magnitude larger than free molecular polarizability.^79,80^ Despite being acknowledged to fluctuate with the coverage rate, this equation can serve as a useful initial reference to screen potential molecules for photosensitizers according to the intended work function.^75,76^ Indeed, this relationship between both properties makes it important to regulate the polarizability of molecules for achieving a desired electrochemical behavior. Through this connection, it can be seen that molecules with higher polarizability tend to facilitate electron injection while those with lower polarizability tend to facilitate hole injection.^76^

**Fig. 5:**
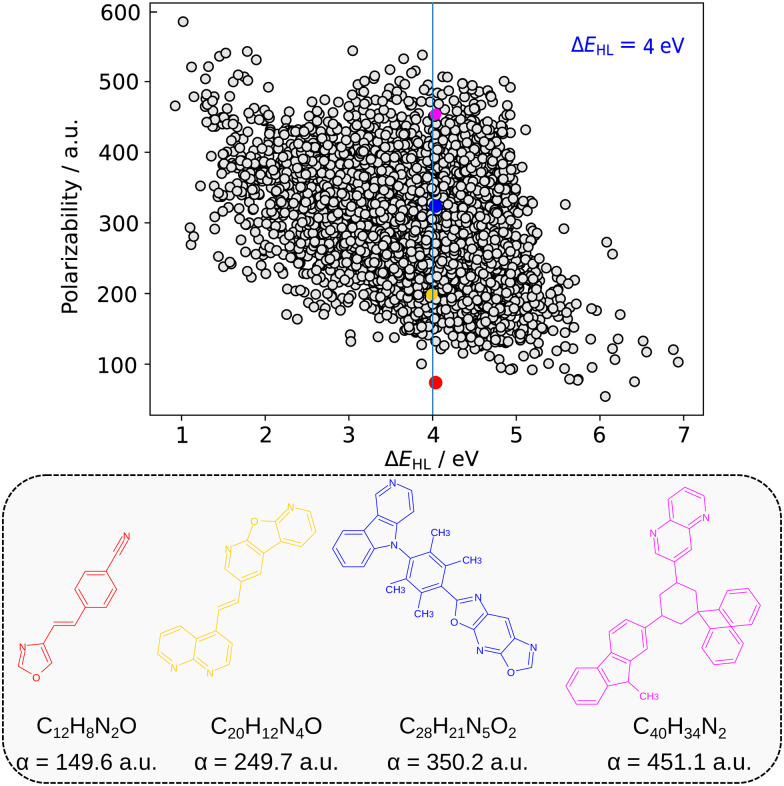
HOMO–LUMO gap and polarizability of the structures in the donor–acceptor (DA) dataset of Xu *et al.*^71^ The maximum of the HOMO–LUMO gap density (4 eV) is marked with a blue line. The four structures corresponding to the four quartiles in the predicted polarizability (within ±0.1 eV) are also shown, together with the predicted values (in a.u.).

In the preceding sections, we have postulated that polarizability and HOMO–LUMO gap are uncorrelated if a large enough subset of the CCS is considered. This law can now be translated to the domain of organic photodetectors: since HOMO–LUMO gap and polarizability are generally independent, it should be possible to design a photodetector with a given detection peak having an arbitrary work function. Alternatively, if matching of electrochemical properties of different systems is the goal, it should be possible to design organic photodetectors with each having arbitrary optical detection windows, yet having the same effect on the work function of electrodes. To demonstrate this statement, we use a dataset generated by Xu *et al.*,^71^ who employed a self-improving Bayesian search to predict possible photodetector molecules in a large subset of CCS. The selection criterion for possible photodetectors was based on both HOMO–LUMO gap and singlet–triplet energy gap, which were evaluated from ground state DFT and TD-DFT calculations, respectively. From all predicted molecules having a donor and an acceptor site (DA structures), we have only selected those cases which have the same atom types as QM7-X molecules (see also in Table 1), leading to a total of 5311 structures. Using the atomic additive model described in Section 3.1, we have estimated the polarizabilities of the selected structures; the plot of the polarizability *versus* HOMO–LUMO gap is shown in the top panel of Fig. 5. Here, one can see that most structures are found in a relatively extended region having Δ*E*_HL_ between 3 and 5 eV and *α* between 200 and 400 a.u., with the possibility to find outliers in all directions around this cluster. In particular, if a high shift in the work function is desired, there appear to be several good candidates with varying optical absorption ranges (see bottom of the graph).

Moreover, our calculations show that the polarizability of these structures can vary by a factor of up to six for specific values of the HOMO–LUMO gap. To demonstrate this flexibility in *α*, Fig. 5 also shows the four molecules corresponding to the four quartiles having a HOMO–LUMO gap of 4 ± 0.1 eV, selected to correspond to the maximum density of data. For this specific HOMO–LUMO gap, polarizability changes between 149.9 a.u. and 451.1 a.u. Taking the tenfold enhancement between the polarizability of the free molecule and the absorbed *α*′ into account and using approximate values of *μ* = 4 D and *d* = 1.5 nm with a full surface coverage, this would mean that changes in work function could range from 0.9 eV to 1.5 eV. These variations are larger than usually achievable by modifications of a semiconductor structure or controlling the surface coverage.^80,81^ Therefore, our analysis shows that work function can be, for practical purposes, freely tailored, even with a very specific design requirement on HOMO–LUMO gap.

This flexibility is also relevant in the task of designing wavelength-selective detectors, which would imply hard constraint on HOMO–LUMO gap. If Δ*E*_HL_ and *α* could not be controlled independently, then optical design restrictions would directly influence the electrochemical behavior. The decoupling of Δ*E*_HL_ and *α* means that the wavelength of detection and the work function can be controlled independently. Fine-tuning the work functions to achieve matching on the metal–organic interface at the electrode is crucial for efficiency. Thus, with the existent “freedom of design” in (Δ*E*_HL_,*α*)-space, we have demonstrated that an efficient detection can be theoretically achieved for any detection wavelength. Alternatively, since the work function can be tailored to match any detection wavelength, it is also possible to design detectors for different detection ranges having equivalent electrochemical properties such as sensitivity, dark current, adhesion behavior as well as any other properties determined by the work function.

## 5 Conclusions

Predictive molecular design is an emerging tool in modern molecular physics and chemistry which heavily relies on the understanding of relationships between key structural and electronic properties. Identifying and explaining correlations between properties necessitates either deep physical understanding or exhaustive data analysis. Herein, we have presented a comprehensive investigation of the intricate interplay between the HOMO–LUMO gap and dipole polarizability – two central properties in designing molecules with tailored optical properties and intermolecular interactions.

Despite the fact that both quantities have their root in the molecular electronic spectrum, understanding their correlation is quite complex. On one hand, the properties are essentially uncorrelated when accounting for a vast chemical space. On the other hand, when examining a small subset of the chemical compound space with similar functionalities, such as homologous series of molecules like oligomeric hydrocarbons, we show that the properties can be observed as being correlated.

To perform a data-driven analysis, we extended the QM7-X database with functional group labels and accurate polarizabilities to explain the physical cause of this phenomenon. Our results demonstrate that the atomic composition has a major role in determining polarizability, while the arrangement of these atoms into chemical functional groups dictates the HOMO–LUMO gap. The physical origin of molecular polarizability was elaborated by studying conceptual models as well as interpreted with the help of a first order linear atomic additive model. Finally, the “freedom of design” arising from the interaction of HOMO–LUMO gap and polarizability was used on the example of organic photodetectors, demonstrating that the electrochemical properties of such molecules can be freely tailored even with specific requirement on the optical properties. The theoretical insights gained from this work can give the basis for expanding the understanding of the relationship between HOMO–LUMO gap and polarizability by incorporating additional descriptors such as molecular size and electronic mobility. Additionally, the unraveled “freedom of design” could be applied to the development of new compounds with tailored optical and electronic properties for use in applications such as organic electronics, sensing or energy harvesting.

## 6 Computational methods

Generally, molecular design is a multi-property optimization problem and requires an exhaustive analysis of diverse structure–property and property–property relationships.^1,6^ In this contribution, we have opted to focus on the two-dimensional property space defined by Δ*E*_HL_ and *α* (*i.e.* (Δ*E*_HL_,*α*)-space), as motivated in the introduction (see Section 1). To calculate the polarizability, two approaches were used: (i) the revised linear additive atomic model of Bosque (introduced in Section 3.1) was utilized for the prediction of polarizability of the organic photodetector candidates in Section 4 and (ii) density functional perturbation theory (for the case studies in Section 3.2 as well as to analyze the QM7-X molecules in Section 3.3). The HOMO–LUMO gap was always obtained from DFT calculations, either by calculating it ourselves or utilizing the values provided in the QM7-X dataset.

### 6.1 Target molecular property space

To perform a purely data-driven study, we utilize the QM7-X dataset^41^ containing 42 physicochemical properties of ≈ 4.2 M (equilibrium and non-equilibrium) organic molecules with up to seven heavy (non-hydrogen) atoms (including C, O, N, S and Cl), spanning a practically important subset of CCS. Accordingly, a subset of QM7-X considering only one equilibrium constitutional isomers and stereoisomers per unique molecular graph is selected for further analysis (≈13 k molecules). In QM7-X, the molecular structures were optimized using the third-order self-consistent charge density-functional tight binding method (DFTB3)^82^ supplemented with a treatment of many-body dispersion/van der Waals interactions *via* the MBD approach.^47,83^ However, for our studies concerning polarizability, *α* was computed directly, employing density functional perturbation theory (DFPT)^33^ by means of the PBE0^84^ functional as implemented in the FHI-aims code^85^ (version 190205). To ensure the transferability of the values, we store the molecular (mean) polarizability (denoted as *α* and simply referred to as *polarizability* in other parts of the manuscript)6
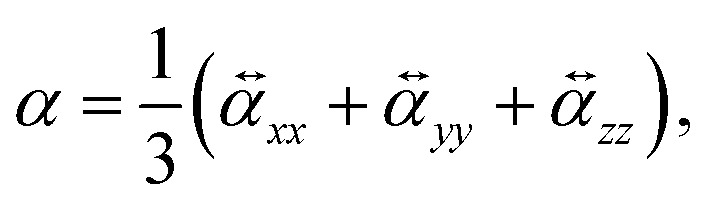
which is independent of the molecular orientation. A second orientation-independent observable, the polarizability anisotropy (Δ*α*) is also often reported, defined as7
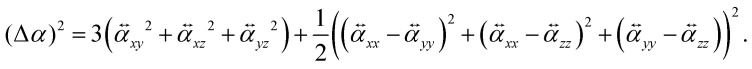
This quantity is mainly used in the description of macromolecules and supramolecular systems, and since our focus is small organic molecules, we don't analyze the anisotropy in this manuscript.

It is known that polarizability is sensitive both to the choice of functional as well as the basis set size.^25,86,87^ To converge our computational setup, the respective mean polarizabilities were compared with the highly accurate values of the QM7b database.^88,89^ This comparison ensures an accurate assessment of the prediction error due to the following two reasons: (i) there is a large overlap between the structures in the QM7b and QM7-X databases, and (ii) QM7b provides highly accurate *α* values obtained at the linear-response coupled cluster singles and doubles (LR-CCSD) level of theory.^35,57^ Then, we computed the polarizabilities of 300 randomly selected structures of QM7b employing the same DFPT computational setup described above. We have found that the PBE0 hybrid functional using the default *light* basis set for all elements amended with three additional functions from the *tight* level predicts *α* with a mean average error of 1.9% and a standard deviation of 1.1% (*cf.* Fig. S1 of the ESI†). The accuracy of our chosen computational setup is higher than to common DFT methods, and slightly better than the 2.84% found by Hait and Head-Gordon^87^ for the PBE0 functional, which can be attributed to the fact that our study is concerned only with organic molecules. Polarizability anisrotropy is predicted with a mean average error of 10.2% with a standard deviation of 5.1%, which is in line with previously reported values.^90,91^ In general, the mean polarizability is slightly underestimated, whereas the anisotropy is almost always overestimated by PBE0.

### 6.2 Molecular classification: functional groups

A workflow has been implemented to identify chemical functional groups from the molecular structure in two steps: firstly, we save the Cartesian coordinates of molecules in a MDL Molfiles format using the standard implementation in Open Babel.^92^ Secondly, Checkmol^93^ is employed to detect the functional groups (204 tags) based on the connectivity tree. In total, 61 unique functional groups were detected for the subset of the ≈13 k QM7-X-molecules,^41^ demonstrating that the dataset covers a considerable sector of CCS (*cf.* Fig. S2 of the ESI†). Since Open Babel predicts valencies only based on the distance between pairs of atoms, the functional group detection scheme is prone to errors for molecules with rare functional groups. Moreover, the functional group definitions of Checkmol have significant overlaps, *e.g.* the molecules detected as alkylamines are also detected as primary amines. To ensure that these shortcomings do not influence our conclusions, we base our analyses only on the subset of the 14 k molecules that have certain functional groups. These groups are chosen to be chemically important, non-overlapping, and each of these categories contain at least 500 entries. The number of structures containing one of these functional groups is 9604. For the analysis in Section 3.3, only molecules containing a single functional group are considered, *i.e.* 1626 entries of our dataset (see Fig. S3, ESI†). Based on these constraints, the following eleven classes of molecules are identified: aldehydes, carbonitriles, dialkyl ether, enol ether, hydrazones, ketones, oximes, primary alcohols and amines, as well as secondary alcohols and amines (see labels in Fig. 4(a)).

## Author contributions

The work was initially conceived by SG, LMS, and AT, and designed with contributions from CM and AT. AT supervised and revised all stages of the work. All authors discussed the results and contributed to the final manuscript.

## Data availability

The dataset used in this work, *i.e.* the subset of QM7-X extended by the DFPT polarizabilities and functional group labels is available on GitHub.^94^

## Conflicts of interest

There are no conflicts to declare.

## Supplementary Material

CP-025-D3CP02256K-s001
