# Supplementary material for: Data-driven tailoring of molecular dipole polarizability and frontier orbital energies in chemical compound space
Source: Phys Chem Chem Phys. 2023 Aug 11;25(33):22211–22. doi: 10.1039/d3cp02256k (PMC10445328; doi:10.1039/d3cp02256k)
Supplement: CP-025-D3CP02256K-s001 [file CP-025-D3CP02256K-s001.pdf]

Electronic Supplementary Material (ESI) for Physical Chemistry Chemical Physics.
This journal is © the Owner Societies 2023

Data-Driven Tailoring of Molecular Dipole Polarizability and Frontier Orbital Energies in Chemical Compound Space

Szabolcs Góger[a], Leonardo Medrano Sandonas[a], Carolin Müller[a], Alexandre Tkatchenko[a,*]

[a] Department of Physics and Materials Science, University of Luxembourg, L-1511 Luxembourg City, Luxembourg
[*] E-Mail: alexandre.tkatchenko@uni.lu

1 Benchmarking the DFPT Polarizabilities

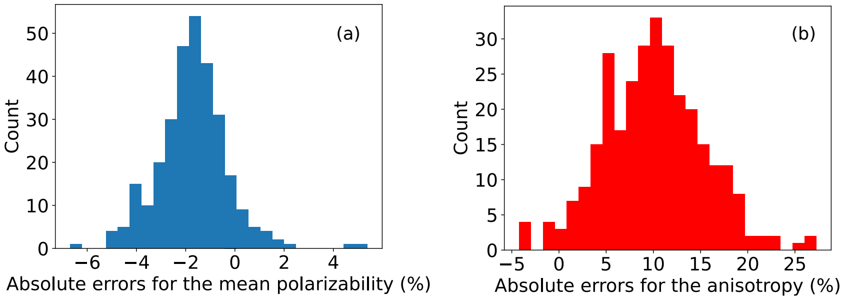

Fig. S 1: Distribution of the relative errors of the mean polarizability (a) and the polarizability anisotropy (b) calculated at DFPT/PBE0 level of theory compared to LR-CCSD reference values.

2 Kolmogorov-Smirnov-distances

To quantify the difference between the distributions of the eleven studied molecular classes in the ΔE_{HL}-α space, the pairwise Kolmogorov-Smirnov-distances (*i.e.*, 55 unique pairs) were calculated between the normalized quantities using the SciPy implementation [1]. An (unnormalized) example for an unique pair is shown in the main article in Figure 1b (α) and 1d (ΔE_{HL}) for non-conjugated aldehydes and primary alcohols. The average distances were found to be 0.81 and 0.40 for the HOMO–LUMO gap and polarizability, respectively.

The Kolmogorov-Smirnov distance for two probability distributions i and j is defined using their individual empirical distribution functions $F(X)$ as

$$D_{ij} = \sup |F_i(x) - F_j(x)| \ , \tag{1}$$

Most commonly, the Kolmogorov-Smirnov distance is used in testing whether the probability distributions i and j have the same underlying distribution [2]. In our case, we calculate this metric for distributions that are known to be different, not for the purpose of a statistical test, but to quantify the distances of the distributions. In this context, the absolute value of the Kolmogorov-Smirnov distance has little practical information, however comparison of the distances confirm that HOMO–LUMO gap depends on the functional groups present, whereas polarizability does not.

3 Frequency of Functional Groups in QM7–X

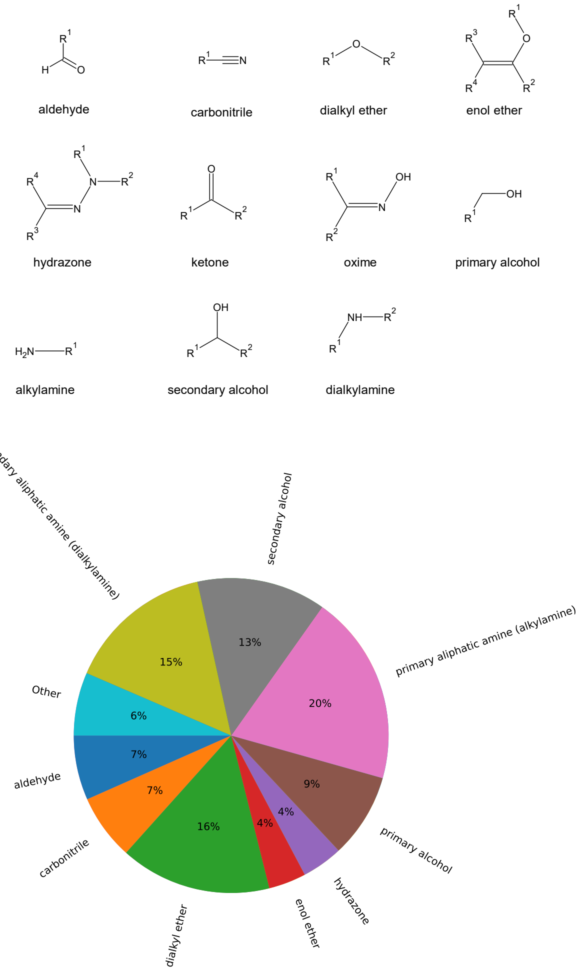

Fig. S 2: Distribution of functional groups in the herein studied subset of 13 k QM7–X-molecules and example molecules for the identified eleven main molecular classes.

4 Dataset Reduction

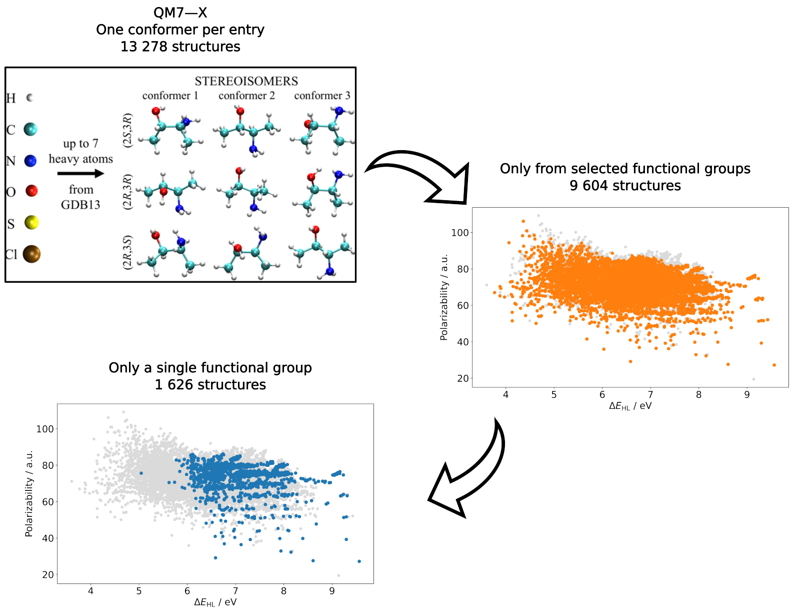

Fig. S 3: Schematic representation of the different data selection we used. Starting from the full QM7–X containing non-equilibrium structures as well as different conformers, we only select a single equilibrium conformer per entry. We then select only those molecules that have functional groups only from our selected list (see Fig. S 2). For analyses where functional group labeling is needed, the structures that only have a single functional group are used.

5 Distribution of Functional Groups in the $(\Delta E_{\mathrm{HL}}, \alpha)$-Space

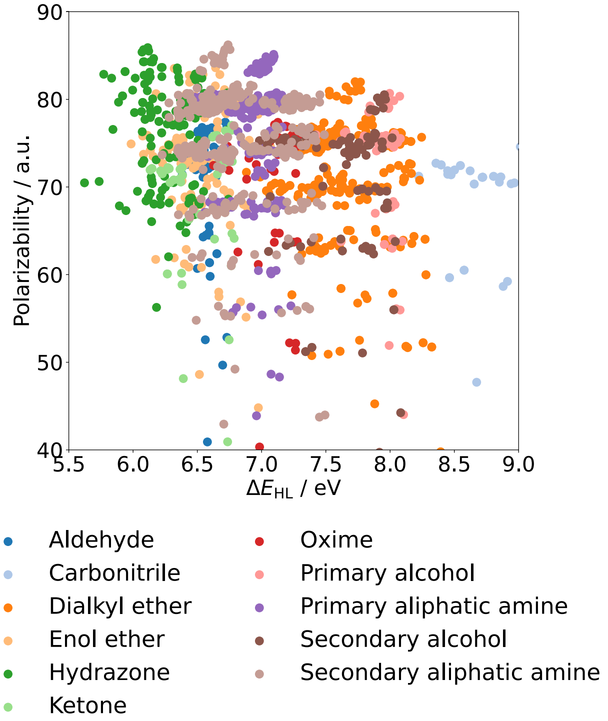

Fig. S 4: HOMO–LUMO gaps and polarizabilities of structures having a single functional group shown in Fig. S 2. The plot shows that the two quantites are uncorrelated as well as that ΔE_{HL} is clustered by functional groups whereas α is not.

6 Structure of the Linear Octenone Isomers

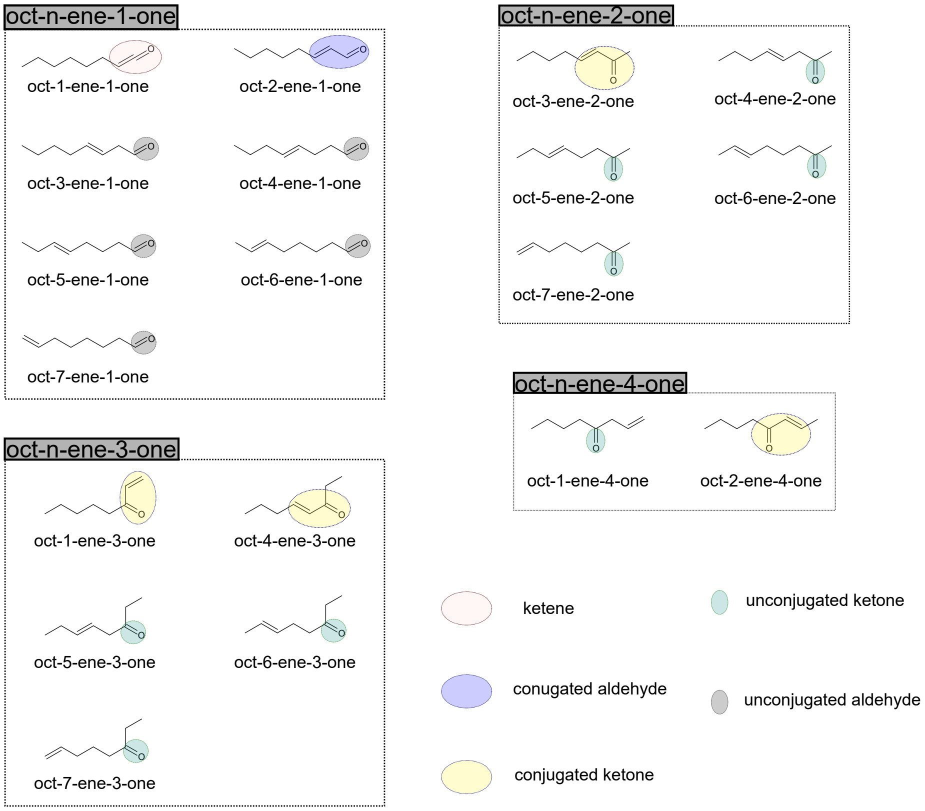

Fig. S 5: Structures of the octenone molecules used in creating Fig. 2 of the main text, with their chemical functionality highlighted.

References

[1] P. Virtanen, R. Gommers, T. E. Oliphant, M. Haberland, T. Reddy, D. Cournapeau, E. Burovski, P. Peterson, W. Weckesser, J. Bright, S. J. van der Walt, M. Brett, J. Wilson, K. J. Millman, N. Mayorov, A. R. J. Nelson, E. Jones, R. Kern, E. Larson, C. J. Carey, İ. Polat, Y. Feng, E. W. Moore, J. VanderPlas, D. Laxalde, J. Perktold, R. Cimrman, I. Henriksen, E. A. Quintero, C. R. Harris, A. M. Archibald, A. H. Ribeiro, F. Pedregosa, P. van Mulbregt and SciPy 1.0 Contributors, *Nat. Methods*, 2020, **17**, 261–272.

[2] M. H. DeGroot, *Probability and Statistics*, Addison-Wesley Pub. Co., 1986.
